# Monitoring wound healing in a 3D wound model by hyperspectral imaging and efficient clustering

**DOI:** 10.1371/journal.pone.0186425

**Published:** 2017-12-07

**Authors:** Mirwaes Wahabzada, Manuela Besser, Milad Khosravani, Matheus Thomas Kuska, Kristian Kersting, Anne-Katrin Mahlein, Ewa Stürmer

**Affiliations:** 1 INRES-Phytomedicine, University of Bonn, Nussalle 9, Bonn, Germany; 2 Department of Translational Wound Research, Centre for Biomedical Education and Research (ZBAF), University Witten/Herdecke, Witten, Germany; 3 CS Department, Technical University of Darmstadt, Darmstadt, Germany; 4 Institute of Sugar Beet Research (IfZ), Göttingen, Germany; Cedars-Sinai Medical Center, UNITED STATES

## Abstract

Wound healing is a complex and dynamic process with different distinct and overlapping phases from homeostasis, inflammation and proliferation to remodelling. Monitoring the healing response of injured tissue is of high importance for basic research and clinical practice. In traditional application, biological markers characterize normal and abnormal wound healing. Understanding functional relationships of these biological processes is essential for developing new treatment strategies. However, most of the present techniques (*in vitro* or *in vivo*) include invasive microscopic or analytical tissue sampling. In the present study, a non-invasive alternative for monitoring processes during wound healing is introduced. Within this context, hyperspectral imaging (HSI) is an emerging and innovative non-invasive imaging technique with different opportunities in medical applications. HSI acquires the spectral reflectance of an object, depending on its biochemical and structural characteristics. An *in-vitro* 3-dimensional (3-D) wound model was established and incubated without and with acute and chronic wound fluid (AWF, CWF), respectively. Hyperspectral images of each individual specimen of this 3-D wound model were assessed at day 0/5/10 *in vitro*, and reflectance spectra were evaluated. For analysing the complex hyperspectral data, an efficient unsupervised approach for clustering massive hyperspectral data was designed, based on efficient hierarchical decomposition of spectral information according to archetypal data points. It represents, to the best of our knowledge, the first application of an advanced Data Mining approach in context of non-invasive analysis of wounds using hyperspectral imagery. By this, temporal and spatial pattern of hyperspectral clusters were determined within the tissue discs and among the different treatments. Results from non-invasive imaging were compared to the number of cells in the various clusters, assessed by Hematoxylin/Eosin (H/E) staining. It was possible to correlate cell quantity and spectral reflectance during wound closure in a 3-D wound model *in vitro*.

## Introduction

Treatment of wounds, which differ in their origin (e.g. traumata or chronic diseases) is a significant medical challenge. During the healing process many different tissue structures and cell lineages are involved [1]. This healing process starts immediately after the moment the tissue was injured [2]. The time span of the healing process is influenced by several external and internal parameters. As reviewed in [1] sedentary cell lineages are triggered by different signaling pathways to proliferate at the wound margin and subsequently invade, establishing a new matrix within the wound area. Depending on the condition of the patient, treatment, circumstances and origin of the wound, this process last one to several weeks or can evolve into a chronic disease.

The constitution of wounds, namely their depth and healing vitality often cannot be assessed extraneously. Due to a rising amount of patients suffering from impaired healing processes, the characterization of chronic wounds is of increasing importance because this knowledge is prerequisite for the adaption of the therapeutic treatment [3]. A wound, bearing a vital wound bed and necrotic wound edges needs a completely different clinical care management compared to a deep and clean, but avital wound. However, the eschars allow no unambiguous assignment to the type of wound. The analyses of biopsies bring more clarity with regard to the diagnosis, however, the sampling operation results in additional local tissue trauma. This situation should be avoided, definitely. In medical research, different *in-vitro* and *in-vivo* approaches were established to investigate processes and biological markers associated with normal and pathologic wound healing.

For a deeper understanding of influencing parameters, hyperspectral imaging (HSI) offers several prospects as an innovative non-invasive investigation method in wound healing research [4, 5]. When light irradiates on an object, it can be either absorbed, transmitted or reflected [6, 7]. Hyperspectral sensors assess reflectance information of an object in several hundred relatively narrow wavebands [8]. Contrary to non-imaging sensors, which assess the mean spectral information of a certain area, hyperspectral imaging provides reflectance spectra for each, spatially resolved image pixel [9, 10]. The resulting HSI data is structured in complex matrices with two spatial x- and y-axes and one spectral dimension (z-axes) with reflectance intensities per wavelength. The spatial resolution depends on the distance between the sensor and the measured object as well as on optical components used in combination with the hyperspectral sensor. Sensing of tissue reflectance in the visible range (VIS, 400–700 nm) and near infrared (NIR, 700–1000 nm) can provide objective information about the phenology, physiology and biochemistry of wound models. In recent years, significant progress in hyperspectral imaging devices have been observed and new hyperspectral sensors for applications from proximal sensing (e.g. hyperspectral microscopy [11]) to air- and space-borne application (e.g. satellite campaigns [12]) launched the marked. In combination with sophisticated data analysis methods from machine learning or data mining, valuable information about biological objects, which could be not assessed by the human eye, are detectable [13, 14]. In the observation of biological systems, reflectance sensing has shown to provide relevant information on e.g. the vitality of ecosystems [15], the health status of crop plants [16] or in assessing quality parameters of food [17] or forensics [18].

Recently, different researches applied hyperspectral techniques to medicine and skin observation. Within this context, previous studies demonstrated that hyperspectral imaging displays a benefit for the examination of human melanoma and their precursors [19]. Gerstner et al. [20] implemented hyperspectral imaging in head and neck oncology for the detection of tumour tissue. Particularly, oncological investigations are reliant on non-invasive examination methods, because it is essential to avoid the damage of tumour tissue to decrease the risk of the generation of metastases. Similar advantages of hyperspectral analysis as an imaging method for the detection of pathophysiological changes in the tissue compartment were shown for the detection of mucosal tumours [21–23]. For implementing hyperspectral imaging into clinical routines, robust findings have to be evaluated by innovative and up-to-date sensing techniques, coupled with advanced data analysis and proper biological interpretation. Following this approach, this stress-less and objective optical sensing technique is a promising tool to evaluate the actual state of injured tissue for the estimation of wound healing progress to enable the adaption of the respective wound management. The advantage of non-invasiveness is undoubtedly. Before studying hyperspectral imaging on elderly patients suffering from impaired wound healing, this *in-vitro* study should give more insights into the spectrum of information provided by hyperspectral imaging and efficient clustering. The obtained results will provide relevant information about a possible correlation between spectral data and wound depth or the vitality of fibroblasts as the representative cell type of the human dermis.

## Materials and methods

**Fig 1:**
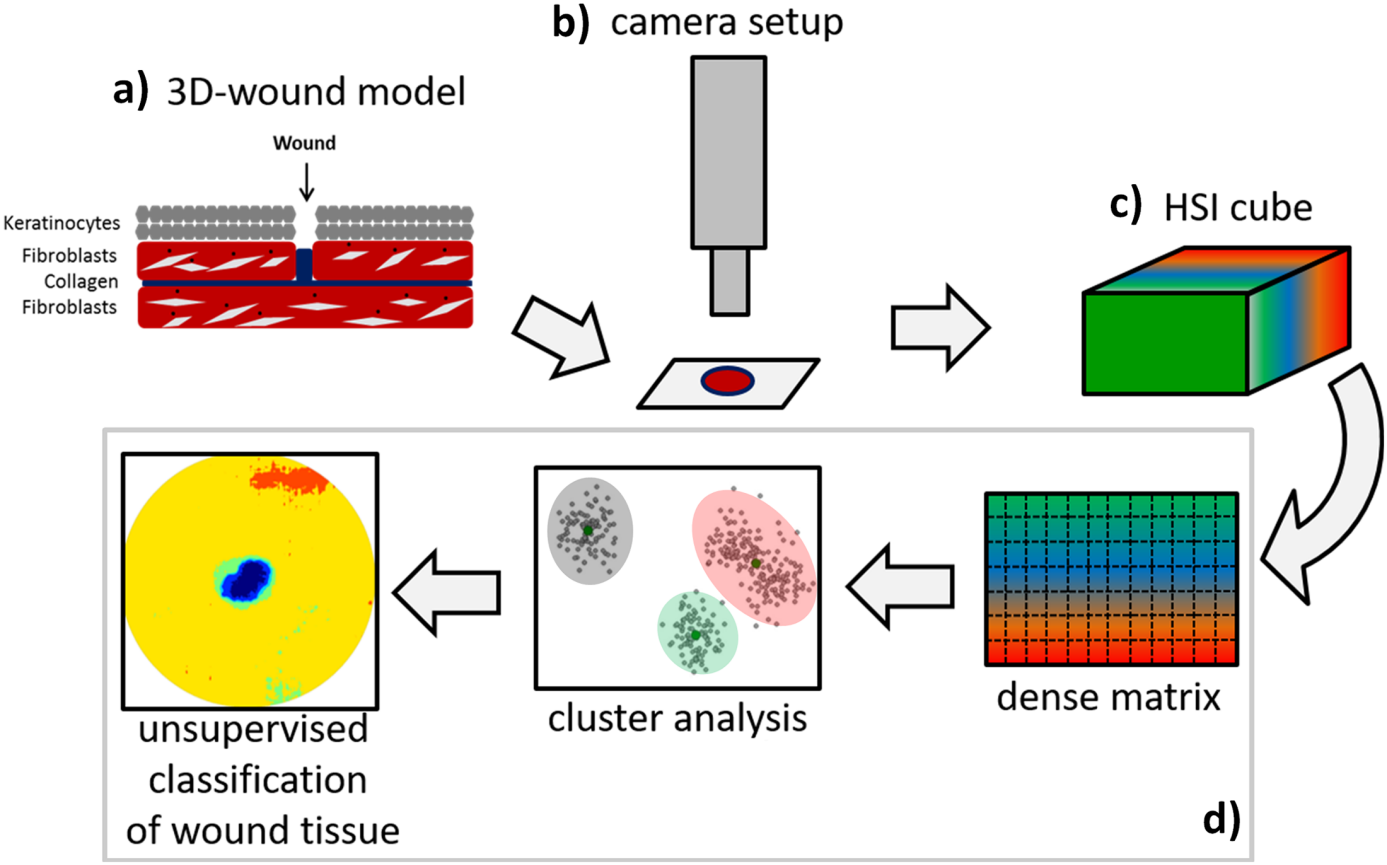
Automated and efficient interpretation of 3D wound models by non-invasive hyperspectral imaging *in vitro*. Experimental design and work flow showing the different steps for monitoring wound healing from hyperspectral imaging data to interpretation using an efficient approach for unsupervised classification of wound tissue based on hierarchical decomposition according to archetypal data points.

In the following of this section we will give a detailed description of the experimental design and work flow from a 3D wound-model to the interpretation of dynamics during wound healing by HSI, as is shown in Fig 1(a)–1(d). The *in-vitro* wound models (a) were measured using a hyperspectral push broom camera (b). The resulting hyperspectral data cubes (c), consisting of spectral information at different spectral bands for each pixel, were then transformed into a dense wavelength×pixel matrix prior to the analysis by an efficient approach for unsupervised classification based on hierarchical decomposition of spectral information (d).

### Cell culture

Human foreskin fibroblasts (CRL2522; ATCC) and human adult low calcium high temperature keratinocytes (HaCat; CLS) were maintained in growth medium containing DMEM (with 3.7 g/l NaHCO_3_, with 4.5 g/l glucose and stable L-glutamine; Merck), 10% fetal calf serum (FCS; Pan Biotech) and were supplemented with 1 ng/ml basic fibroblast growth factor (bFGF) and epidermal growth factor (EGF) (Pan Biotech). The cells were cultured under humidified conditions with 10% CO_2_.

### Processing of acute and chronic wound fluid (AWF/CWF)

For collection of AWF and CWF approval was given by the ethics comittee of the University of Witten/Herdecke (39/2007). Written informed consent was obtained from all patients for sample collection. CWF was harvested from patients suffering from a third degree chronic sacral decubitus ulcer. The criteria implied the persistence of the wound for at least 6 weeks and the exclusion of patients who received vacuum therapy or surgery, previously. CWF was collected by administration of occlusive dressings for 24 hours (h). AWF was accumulated from patients, who underwent abdominoplasty. The collection began 8 h post operation and lasted 8 h. After collection, both fluids were immediately centrifuged at 4000 rpm for 5 min, the supernatant was sterile filtered and stored at -80°C. Wound fluids were pooled from 5 patients.

### Three-dimensional (3D) wound model system

#### Preparation of the collagen gel matrix

Fibroblasts were harvested by trypsinization and counted automatically with the NucleoCounter (Chemometec). After centrifugation at 1200 rpm for 5 min, the cells were resuspended in culture medium to a final concentration of 1 x 10^6^ cells/ml. All ingredients for the collagen matrix were stored on ice before the construction was started and mixed in the exact order listed in the following example for the assembly of at least 6 layers: 1040 *μ*l 10x DMEM (Biochrom), 208 *μ*l 1 M NaOH (Merck), 4,4 ml dH_2_O (ChemSolute), 260 *μ*l 7.5% NaHCO_3_ (Carl Roth), 2.6 ml fibroblast growth medium, 4.42 ml (9 mg/ml) rat tail collagen I (Corning; final gel concentration of 2.25 mg/ml). The pH of the solution was adapted to 7.4 with 1 M NaOH and 3.25 ml cell suspension (with a final concentration of 5 x 10^5^ cells/layer) was finally added. After gentle trituration, 2.5 ml solution per layer was transferred to a 6 well culture plate. The collagen polymerized by incubation at 37°C in a cell culture incubator, containing 10% CO_2_ for 30 min. Finally, the gels were detached from the sides of the well to enable the contraction of the matrix within 8 days. The media were changed daily.

#### Construction of the 3D wound model

The contracted collagen matrix was gently transferred to a 12 well culture plate and keratinocytes were added on top of the gel with a final concentration of 1 x 10^6^ cells per one 3D model system. The keratinocytes were allowed to adhere and grow to confluency in normal growth medium for another 4 days. On day 12 after initial preparation, the collagen matrix bearing the keratinocytes became wounded using a 2 mm circular biopsy punch. This layer was placed carefully onto the counterpart gel matrix without biopsy, which was moistened with 200 *μ*l adhesive solution shortly before. This connective “glue” consisted of the same collagen matrix solution, however, the fibroblast growth medium was additionally supplemented with 10% artificial or chronic wound fluid, respectively. The 3D models were incubated at 37°C in the cell culture incubator for 30 min without medium to facilitate the polymerization of the adhesive solution and to avoid shifting of the two single layers. Finally, the models were cultured in growth medium supplemented with 10% AWF or CWF. Under control conditions any supplements were added. Medium was changed every second day and the cultures were incubated for 5 or 10 days, respectively, at 37°C in a 10% CO_2_-enriched atmosphere.

### Hyperspectral measurements

Spectral reflectance was measured with a hyperspectral push broom camera (Spectral camera PFD V10E, Spectral Imaging Ltd., Oulu, Finland). The spectral range of the PFD V10E spectral camera was 400 to 1000 nm with a spectral resolution of 2.73 nm. The 30 *μ*m sensor results in 1300 pixel per line with a sensor pixel size of 0.0074 mm. The spectral camera was installed on a magnification system (Z6 APO, Leica, Wetzlar, Germany) aligned to a motorized XY-table (Prior Scientific GmbH, Jena, Germany). The software SpectralCube (Spectral Imaging Ltd.) was used to regulate camera settings and to acquire hyperspectral images. Images were taken with spectral binning 1 and spatial binning 1 to obtain the highest possible resolution. Frame rate and exposure time were adjusted to the object. Each specimen was transferred on a low reflectance dish (Brightic Black Dish©, Nagymaros, Hungary) and placed nadir under the camera on the xy-table. An area of 2.5 *cm*^2^ was scanned per image and individual/treatment. Samples were magnified 7.3 x, resulting in an image pixel size of 7.5 *μ*m. For homogeneous illumination, light lines (Schott AG, Mainz, Germany) with 150 watt halogen tungsten bulbs connected via a non-absoring fiber (DCR^®^ Light Source EKE, Polytec, Waldbronn, Germany) were placed horizontally to the field of view of the hyperspectral camera. Detailed information on the technical set-up can be found in [11, 24]. Hyperspectral measurements were performed in a dark room on 0/5/10 days *in vitro*.

### Data processing

Reflectance of the 3-dimensional wound model samples was calculated by normalizing hyperspectral images to the reflection of a barium sulfate white reference standard (Zenith Polymer Target, SpereOptics GmbH, Uhldingen, Germany) and to a dark current measurement of the signal to noise ratio of the sensor chip after closing an internal shutter. Normalization was performed using ENVI 5.1. + IDL 8.3 (ITT Visual Information Solutions, Boulder, CO). Before further analysis, the Savitzky-Golay filter with a a fifth degree polynomial and seven supporting points to the left and right, each, was applied for smoothing the signals [25]. Because reflection data was noisy at the extremes, only values from 450 to 790 nm were considered for further analysis.

### Automated and efficient interpretation of hyperspectral data

A fast approach for clustering the spectral signatures was applied to identify characteristic hyperspectral representatives of different tissues. Unlike supervised approaches, where the classes are known and an appropriate number of examples for each class is available in order to learn the classifier, in this case no a-priori information or training data for each class was available. The approach is based on an efficient hierarchical decomposition of spectral information, as given by [26] and determines a clustering of the data in form of a tree. That is, in each iteration it first computes a 1D projection of the data and applies a splitting criterion by minimizing the weighted variance as much as possible. The procedure is then recursively applied to each sub-tree until a minimum size of the leaf node (number of signatures) or a predefined depth is reached. In a pruning step the number of clusters is further reduced by additionally computing a clustering on the means of each leaf.

More precisely, following random projection trees [27], a tree decomposition is computed, but using a 1D FastMap projection (due to [28]) in each iteration in order to split the data. The goal of FastMap is to project the data points, in this case the columns of the data matrix ***X***^*m*×*n*^, onto a line between two points $\vec{a}\in X$ and $\vec{b}\in X$ (called pivot points) using the given distances between the points. The process of choosing the pivot points can be summarized in tree steps

1. Pick any data point $\vec{a}\in X$
2. Let $\vec{b}$ be the farthest point from $\vec{a}\in X$
3. Let $\vec{a}$ be the farthest point from $\vec{b}\in X$.

For this, the Euclidean distance between the points, which is defined as

$$\begin{array}{c} \text{Dist}\left(\vec{a},\vec{b}\right)=\sqrt{\sum_{i}{\left(a_{i}-b_{i}\right)}^{2}} \end{array}$$ (1)

was employed.

The projections are computed using the law of cosines as well as the current distances to the last two pivot objects:

$$\begin{array}{c} Proj\left(\vec{x}\right)=\frac{\text{Dist}\left(\vec{a},\vec{x}\right)+\text{Dist}\left(\vec{a},\vec{b}\right)-\text{Dist}\left(\vec{b},x\right)}{2\text{Dist}(\vec{a},\vec{b})}. \end{array}$$ (2)

As the pivot points correspond to the most extreme points in the dataset (or subtrees), this actually computes a decomposition according to the archetypal data points [29]. Therefore, this efficient approach can be called *Extreme Hierarchical Clustering* (XHC).

XHC determines the clustering by first transforming each hyperspectral cube into dense matrix ***X***^*m*×*n*^, where the columns denote the hyperspectral signatures and rows the wavelengths. Starting with an empty tree XHC repeatedly search for the best *Rule* for a node according to some splitting criterion such as the weighted variance along the 1D FastMap dimension. More precisely, it computes the values in $Proj(\vec{x})$ generating the list *s*_1_ ≤ *s*_2_ ≤ … ≤ *s*_*n*_ and finds a point *i* that minimizes

$$\begin{array}{c} c_{i}=\frac{1}{i}\sum_{j=1}^{i}{\left(s_{j}-\mu_{1}\right)}^{2}+\frac{1}{n-i}\sum_{j=i+1}^{n}{\left(s_{j}-\mu_{2}\right)}^{2}, \end{array}$$ (3)

with *μ*_1_ denoting the mean for all points *s*_*j*_ ≤ *s*_*i*_ and *μ*_2_ the mean for the points *s*_*k*_ > *s*_*i*_. The splitting rule is set to

$$\begin{array}{cc} & \text{Rule}\left(\vec{x}\right)≔Proj\left(\vec{x}\right)\leq \theta \;\text{with}\;\theta =\left(s_{i}+s_{i+1}\right)/2. \end{array}$$ (4)

Next, the examples ***X*** in the node are sorted into ***X***_*s*_ (success) and ***X***_*f*_ (failure) according to the *Rule*. For each split, the procedure is recursively applied, obtaining subtrees for the respective splits. The splitting was stopped if a minimum number of examples in a leaf node or a predefined depth is reached. In a second step, a post pruning process via Kmeans clustering was applied on the mean observations for each leaf. This gave the final representatives for the hyperspectral images. To deal with massive hyperspectral data, one can easily parallelise the approach: run the tree decomposition on the subsets of the data separately, collect the means of the leafs for each subset in a matrix ***Y***^*m*×*l*^ and compute the final representatives using Kmeans clustering on the matrix ***Y***. In a third step and finally, all signatures of the images were classified to one of the representatives computed in the previous step.

### Microscopy

All tissue samples were fixed in 4% paraformaldehyde followed by paraffin embedding using standard technique. Sections were prepared with 10 *μ*m thickness. The slices were deparaffinized and rehydrated by ethanol/xylene washing steps before usage. Standard hematoxylin and eosin staining was performed for the histological examination of wound healing. Cell migration, proliferation and apoptosis were investigated by immunohistochemical stainings for Ki67, C-X-C motif chemokine receptor 4 (CXCR4) and Caspase 3 (Cas3). For the detection of Ki67 and CXCR4, the slices were heated in 10 mM citrate buffer pH 6.0 or Tris/EDTA pH 9.0, respectively, cooled down to room temperature (RT) and transferred to 1x phosphate buffered saline (PBS). The sections were permeabilized and blocked with permeabilization buffer containing 1x PBS, 1% bovine serum albumin (BSA; Carl Roth), 0.1% Triton X100 (Merck) and 0.1% Tween 20 (Carl Roth) for 1-2 h at RT. The incubation of the primary antibodies were performed in blocking solution comprised of 1x PBS, 1% BSA, 0.1% Triton X100, 0.1% Tween 20 and 5% goat serum (Pan Biotech) protected from light in a humidified chamber at 4°C over night. After washing with PBS, the secondary antibodies were incubated in blocking solution for 1-2 h at RT, followed by three washing steps with PBS and embedding in Roti^®^-Mount FluorCare DAPI (Carl Roth). The following antibodies were used: Mouse monoclonal Anti-CXCR4 antibody (3 *μ*g/ml; Abcam), mouse monoclonal anti human Ki-67 Antigen Clone MIB-1 (1:50; Daco), rabbit polyclonal anti-Caspase 3 antibody (1:150; Abbexa), Alexa Fluor^®^488 goat anti-mouse IgG (H+L) (1:500; Life Technologies/Invitrogen) and Alexa Fluor^®^568 goat anti-rabbit IgG (H+L) (1:500; Life Technologies/Invitrogen).

### Cellular characterization of spectral clusters

Three consecutive sections from model systems were illustrated by using fluorescence microscopy (Leica DMI4000B). The upper and lower part of the tissue sections were separated in 5 regions of interest (ROI), which covered the wound base and two regions of equal size next to the wound base (wound margin). Areas, depicting predominantly a specific spectral cluster were determined. The spectral signatures were analyzed by the quantification of H/E stained cells referred to the hyperspectral reflectance.

### Statistics

To evaluate statistical relevance all data were analyzed with one-way analysis of variance (ANOVA) and Sidak’s multiple comparisons test by using GraphPad Prism 6 (* p ≤ 0.05; **p ≤ 0.01; *** p ≤ 0.001). Error bars indicate the standard error of means (s.e.m.). N = number of independent experiments, n = the number of data points.

## Results

For the main experiment the parallel setting was used by first computing the hierarchical decomposition of each image, which allowed a fast and memory efficient clustering of the dataset with a total size of approximately 12 GB. To get the hierarchical decompositions XHC required only 3.5 minutes on a standard Intel SixCore machine with 3.2 GHz and 16 GB main memory. Before learning the clustering, the masked areas, consisting of background and some parts covered with fluid (with maximum reflectance values beyond 1) were excluded. To get the representatives, images of day 0 and 5 were used. Cluster assignments were computed in a second step for all signatures and images.

**Fig 2:**
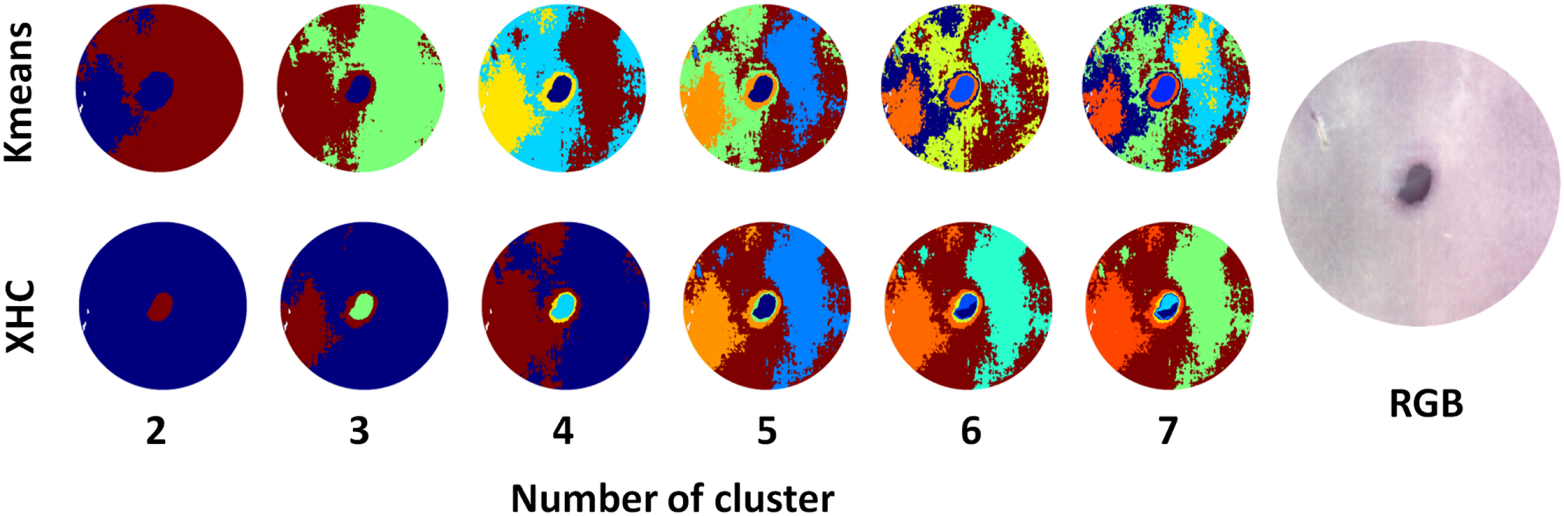
Comparison for Kmeans and XHC for different number of clusters. With increasing number of clusters, Kmeans resulted in more clusters around the wound as this area represented the major part of the tissue. XHC further highlighted the middle part of the images, which was the result of a hierarchical decomposition of the signatures. This allowed a better investigation and comparison of the wound tissue and healing progress.

To compare XHC with Kmeans clustering directly, experiments with changing number of clusters *k* on an example image were run, as shown in Fig 2. With increasing number of clusters, Kmeans resulted in clusters around the wound, representing the major part of the tissue. XHC further highlighted the middle part of the samples. To get the clusterings, the example image was first transformed to a dense matrix, after removing the masked background, the resulting matrix consisted of more than 40 Mio entries. In all settings Kmeans learned until convergence or if the maximum iterations *niter* = 1000 was reached, whereas for the hierarchical decompositions the maximum depth was set to 10. XHC required only a fraction of time to get the decomposition, compared to the standard approach. For instance, for *k* = 7 clusters Kmeans converged after about 10 minutes (average time over several reruns), whereas XHC required under 15 seconds for the computation of the decomposition only.

**Fig 3:**
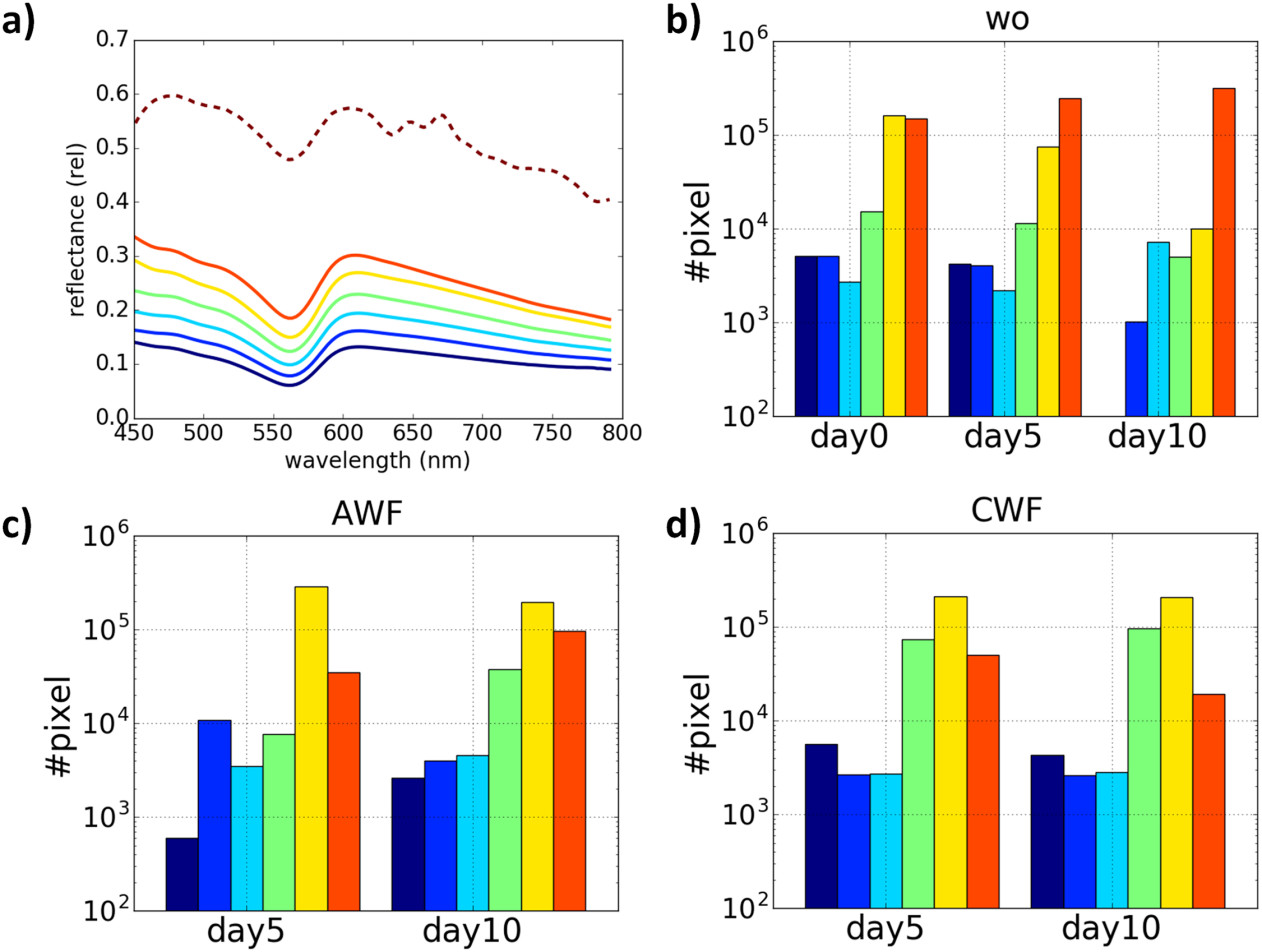
Representative spectra of 3-dimensional wound models. (a) Cluster representatives after computing of XHC for the hyperspectral image data for. The number of cluster was set to *k* = 7 that could reflect biological processes in this cell culture system. The corresponding signatures represented different regions of the tissues. The dotted line was produced by a part of the tissue covered with fluid resulting in overexposure during the measurement. (b-c) The quantification of pixel densities per cluster. The y-axis is shown in log scale. AWF and CWF induced no significant differences in pixel densities compared to the control situation, however, the dark blue cluster was absent after 10 days *in vitro* without any supplements.

**Fig 4:**
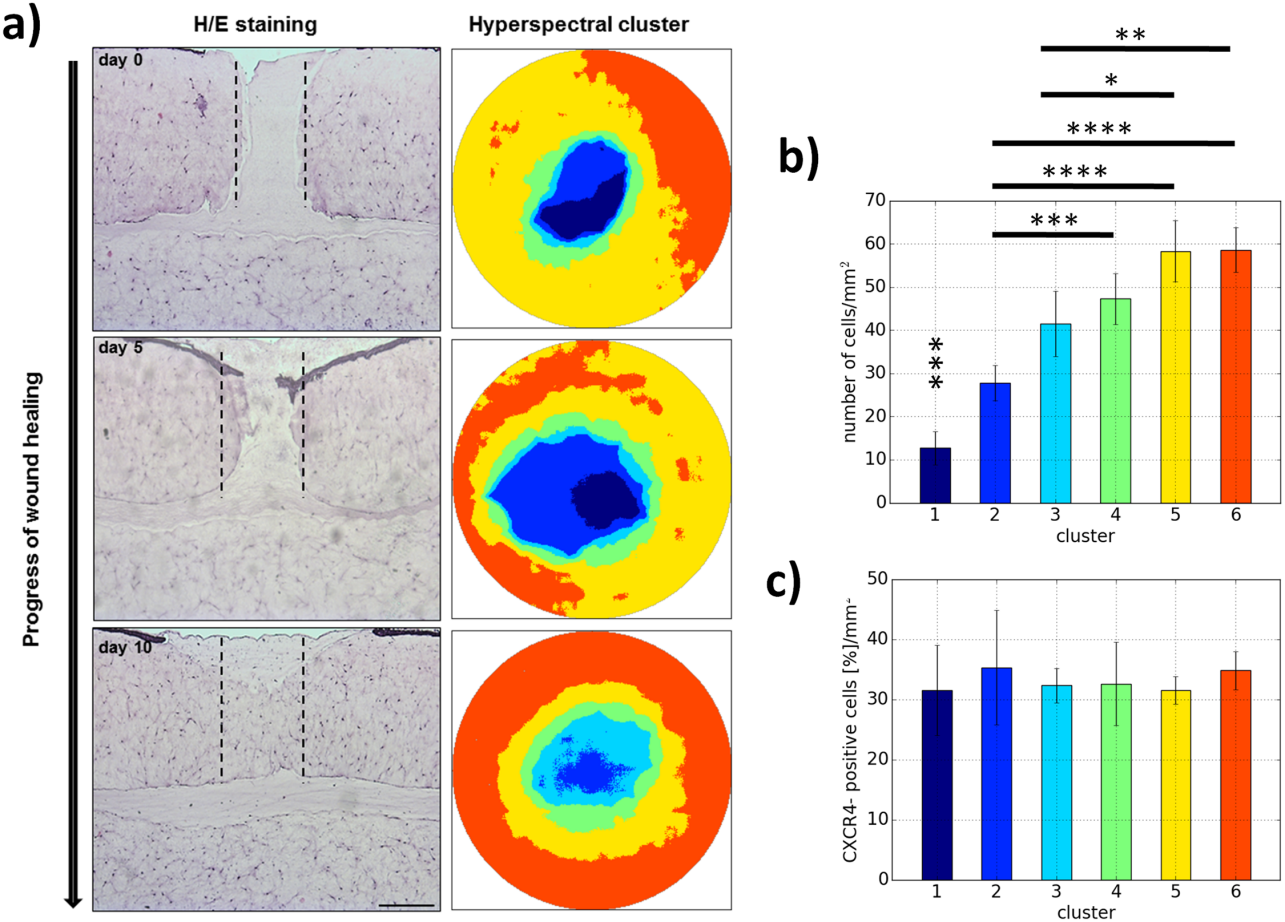
Histological classification of hyperspectral cluster means in relation to wound healing processes. (a) H/E staining was performed to monitor the wound healing progress morphologically over a specific time period *in vitro*. The different stages were presented as false color zoomed images of the hyperspectral clusters for the wounds. (b) The quantification of the cell number in different regions of interests revealed the first time a correlation between spectral reflectance and cell quantity in the tissue. (c) Immunohistochemical investigation of CXCR4, a marker for migratory cells, determined no correlations to reflectance data. Additionally, the cells generating the characteristic hyperspectral signature did not correspond to Caspase 3-expressing apoptotic cells (not shown). Scale bar: 250 *μ*m.

Figs 3 and 4 summarize the results for the main experiment on the entire dataset. For interpretation only the area of 5 mm diameter for each image was considered and results for clustering with *k* = 7 are shown. Fig 3(a) shows the corresponding cluster representatives, representing different parts of the tissue. On the one hand the spectra represented different regions of the discs and on the other hand biological processes in the culture system, which had to be assigned in more detail. The dotted line was based on external fluid. Additionally, the number of pixels per cluster was quantified and compared between the different conditions (Fig 3c and 3d). In wound models, which were cultured in presence of AWF or CWF, the dark blue cluster was continuously detected. Additionally, the yellow spectrum was approximately 10-fold increased under both conditions compared to the control after 10 days (Fig 3c, 3d vs. 3b).

After hyperspectral scanning of the 3D wound models was performed, the discs were fixed immediately and frontal sections were made to monitor the wound healing process to gain further information about the origin of the reflectance signatures (Fig 4). Via hematoxylin and eosin staining, it was possible to reproduce the migration of dermal fibroblasts towards the wounded area and the refilling of the wound bed with proceeding time *in vitro*, as illustrated in Fig 4(a) bottom row. These phenotypic changes were observed by hyperspectral imaging, which depicted a specific spatiotemporal cluster distribution, as shown in Fig 4(a) upper row. The quantification of the number of cells in various regions of interests per cluster, revealed the first time a significant correlation between the quantity of cells and the reflectance data (Fig 4b). Subsequent immunological analysis of the expression of specific markers for migration (Fig 4c) and apoptosis (not shown) evaluated that the reflectance intensity per wavelength was not due to the expression of CXCR4 or caspase 3, respectively. The possibility to exclude apoptotic cells as the responsive factor for the alterations in reflectance pattern with ongoing time, is of high interest with respect to clinical applications. However, the particular cellular factors generating this cluster are not specified yet and still under investigation.

To summarize the results, the approach presented in this study leads to more descriptive clusters, is memory efficient, easy-to-parallelize and allows for decompositions in a fraction of time compared to standard algorithm. Scalability of automated analysis is of great importance in order to get optimal results and fast insights into massive datasets, especially in cases where no prior information is available or difficult to acquire. This data mining approach on hyperspectral images of a 3D wound model provided relevant information on correlation between spectral data and fibroblast dynamics during wound healing as the representative cell type of the human dermis.

## Discussion

Hyperspectral imaging (HSI) can be implemented as a non-invasive optical sensing technique to characterize skin diseases [30]. Within this context, HSI creates a three-dimensional data cube of spectral and spatial information of a biological tissue. Each image pixel contains the spectral information, in the visible and near-infrared range. Compared to other optical systems (such as RGB, laser based systems or MRI), HSI comes along with several main advantages: i) a passive, non-invasive measuring procedure without negative effects on the specimen, ii) high spatial resolutions and iii) spectral resolution beyond the visible range.

The lack of invasiveness as well as a spatial evaluation by HSI is a crucial advantage in human medicine and wound healing monitoring compared to performing biopsies for characterization. Histological analysis of a biopsy often indicate an unspecific inflammation without any further information of cell density, vitality or necrosis. A biopsy represents only a selected region of the surface, however, the tissue state may differ in other areas of affected skin. Finally, the biopsy causes pain and may worsen scare development of a patient. Here HSI can substitute or support common approaches.

Due to the complexity and information richness of the data obtained, HSI has shown the potential to investigate and characterize phenotypic changes in human or plant systems. However, beside the above mentioned advantages, the interpretation of HSI data is demanding and powerful analysis routines are necessary.

Diverse studies were performed to investigate potential applications of HSI for medical diagnostic of tissue status and for monitoring wound healing in a clinical setting. It was demonstrated, that hyperspectral data assess the concentration of oxyhemoglobin and deoxyhemoglobin in diabetic foot ulcers as wells as burn injuries and thereby give information about the oxygen saturation [31–37]. These values were used to predict diabetic feet at risk of ulceration, the potential to heal or the risk of the development of chronic wounds [38]. The evaluation of specific absorption maxima of skin chromophores enabled the investigation of cancer progression, blood flow, microcirculation and reepithelialization [19, 30, 36, 39–41]. However, the interpretation of the reflection data was initially not based on the comparison of specific spectral reference signatures but on the retrospective assessment of light reflectance and the visual observation of tissue condition with or without pathophysiological development.

The present study focused on the investigation of HSI in relation to the composition of the tissue on the cellular level. For this intention, a simple 3-dimensional wound model comprised of human fibroblasts embedded in a collagen matrix and keratinocytes on the surface as representatives of the most important skin cells was implemented. Based on this model the healing process was mimicked *in-vitro* with the advantage to exclude possible influence on the results of the spectral reflectance measurement by the aforementioned absorption of incident light by chromophores or the oxygenation status. The results of the reflectance cluster can be directly referred to fibroblasts and keratinocytes embedded in collagen type 1. Following hyperspectral imaging at different time points of wound healing, the tissue was fixed and investigated histologically and immunohistochemically. It was possible to transfer cluster means of the HSI data to a definite region of the wound and to perform standardized cell quantification in the identified regions of interest (roi). The quantification of the hematoxylin-eosin staining revealed a correlation between a spectral cluster and the number of fibroblasts. Under control conditions, the dark-blue cluster with a mean cell density of 12.7±3.9 cells/mm2 disappeared, due to an increased amount of migrated fibroblasts during wound healing. Concomitantly, the red cluster (cell density: 58.6±5.2 cells/mm2) was slightly elevated in the wound, presumably combined with a reduction of the cell number in the periphery of the wound. The cluster pattern of AWF at day 10 was similar to day 0 in the control situation (Fig 3c vs. 3b). This observation combined with the persisting presence of the dark blue cluster at day 10 in AWF and CWF treated cultures indicates a delayed wound healing. The difference in the hyperspectral clustering during the healing process under CWF challenge was a decrease of the red pattern, representing a fibroblast-rich area. This could be assigned to an impairment or even stagnation of wound healing. Immunohistochemical staining to CXCR4 demonstrated that these cells were actively migrating towards the wounded area. The spectral reflectance was not affected by CXCR4 expression.

Furthermore, it can be concluded that information about the metabolism of the fibroblasts can be extracted from HSI data. Due to the lack of chromophore absorption, it can be possible to determine the spectral signature of the cytochromes b/c, which are located on the membranes of mitochondria and involved in the ATP-synthesis. The spectral cluster is dependent on the redox state of these hemeproteins and thus represents the metabolic activity of cells located in different regions of the wound [5]. The evaluation of the metabolic state and a link to spectral profiles enables to draw conclusions regarding the vitality of the tissue.

In a next step, further analysis will be performed to confirm these results and to get more insight into the interpretation of HSI data on the cellular and molecular level in detail. With this extended information pool achieved by using HSI as a new diagnostic tool, the clinician will be able to specifically adapt the therapeutic management to improve the outcome of wound healing or prevent the aggravation of diseases, respectively. Statements relating to cells promoting wound healing appear quite close. Furthermore it is expected, that due to the current progress in sensors and machine learning technologies, new and powerful tools for human medicine are provided. These may be easy to use hand held sensors with integrated analysis routines for an immediate decision support.

## Conclusion

This study revealed a correlation between cell quantity and spectral signatures from cluster means during wound closure in a 3D wound model *in vitro*. Additionally, it has been elucidated that the reflectance spectrum depends on the metabolic activity of the cells of interest. Thus, hyperspectral imaging can serve as a proxy or alternative for invasive tissue sampling and analysis. Methods for interpretable and sparse decompositions of hyperspectral information could give additional insights into the data, allowing for an identification of interesting hyperspectral characteristics in terms of important spectral ranges and their dynamics over time, for example. Further analysis targeting the specification of the cell condition and behaviour as well as matrix composition during wound healing in relation to the spectral signature will provide deeper insights into the system.
